# Phytotoxic Compounds Isolated from Leaves of the Invasive Weed *Xanthium spinosum*

**DOI:** 10.3390/molecules23112840

**Published:** 2018-11-01

**Authors:** Zhuogeng Yuan, Xiangwei Zheng, Yu Zhao, Ying Liu, Shixing Zhou, Caixia Wei, Yunxia Hu, Hua Shao

**Affiliations:** 1 Chemistry and Environment Science School, Yili Normal University, Yining 835000, China; yzg821871139@outlook.com; 2 Engineering Research Center of Modern Preparation Technology of TCM, Ministry of Education, Shanghai University of Traditional Chinese Medicine, Shanghai 201203, China; zhengxwsh@hotmail.com; 3 Bioscience and Geosciences School, Yili Normal University, Yining 835000, China; 2001zhaoyu@sohu.com (Y.Z.); zylyzhlily@126.com (Y.L.); 4 Key Laboratory of Biogeography and Bioresource in Arid Land, Xinjiang Institute of Ecology and Geography, Chinese Academy of Sciences, Urumqi 830011, China; zhoushixing16@mails.ucas.ac.cn (S.Z.); weicaixia16@mails.ucas.ac.cn (C.W.)

**Keywords:** phytotoxicity, allelopathy, bioactive compounds, natural herbicide, *Xanthium spinosum*

## Abstract

The aim of this study was to identify bioactive compounds from leaves of the invasive plant *Xanthium spinosum* and assess their phytotoxic activity. Activity-guided fractionation led to the isolation of 6 bioactive compounds: xanthatin (**1**), 1α,5α-epoxyxanthatin (**2**), 4-epiisoxanthanol (**3**), 4-epixanthanol (**4**), loliolide (**5**) and dehydrovomifoliol (**6**). Of them, compounds **2**–**6** were isolated from the *X. spinosum* for the first time. The structures of **1**–**6** were elucidated on the basis of extensive NMR studies and ESI-MS measurements as well as comparison with literature data. All of compounds were evaluated for their phytotoxic activity. Among them, compounds **1**–**4** exhibited stronger activity on 2 receiver plants compared with the other 2 compounds, with xanthatin (**1**) being the most potent compound, which suppressed root growth of the dicot plant *Amaranthus retroflexus* by 32.5%, 39.4%, 84.7% when treated xanthatin (**1**) at 5, 20, and 100 µg/mL, while for the monocot plant, root growth was inhibited by 14.7%, 28.0%, and 40.0%, respectively. Seedling growth was nearly completely inhibited when the concentration of xanthanolides increased to 500 µg/mL, whereas there was still some seedling growth when loliolide (**5**) and dehydrovomifoliol (**6**) were applied at the same concentration. Dehydrovomifoliol (**6**) did not negatively affect seedling growth of *P. annua* at all tested concentrations, and root length was still 42.0% of the control when the highest concentration 500 µg/mL was used. This is the first report of the phytotoxicity of 1α,5α-epoxyxanthatin (**2**), 4-epiisxanthanol (**3**) and 4-epixanthanol (**4**). These compounds have the potential to be utilized as natural herbicides, especially 4-epiisoxanthanol (**3**), which exhibited significant selective activity between the dicot and monocot plants. On the other hand, whether these bioactive substances serve as allelochemicals to facilitate the invasion success of *X. spinosum* needs to be further studied.

## 1. Introduction

Exotic plant invasion poses serious threat to the conservation of ecosystems due to its negative impacts on local plant community by displacing native plant species or inhibiting the establishment of new individuals [1]. Exotic plants are often found to perform better in their invaded ranges than in their native ranges, and a number of hypothesis have been proposed attempting to elucidate the mechanism causing this phenomenon, including enemy release, enhanced mutualist, novel weapons, accumulation of local pathogens, etc. [2,3,4]. Among them, the “novel weapons hypothesis” suggests that allelopathy may play an important role in the invasion process of exotic plants via the production of novel allelochemicals, which presumably possess potent phytotoxic effect on native species due to the lack of coevolution [4,5,6]. Allelopathy refers to any direct and indirect harmful or beneficial effect by one plant on another through the production of chemical compounds that release into the nearby environment [7]. There have been a number of reports on the possible involvement of allelopathy in the invasion success of some exotics; however, the “novel weapons hypothesis” has been challenged ever since it was proposed due to the complicated mechanism of allelopathy [8,9,10,11].

China is one of the countries that suffer severe consequences caused by biological invasions. Lots of invasive species belong to the *Asteraceae* family, possibly due to their ability of producing a variety of secondary metabolites with various biological activities [12]. Among the *Asteraceae* plants, *Xanthium spinosum* L., commonly known as spiny cocklebur, is an herbaceous annual weed in the genus *Xanthium* which includes 25 species that originates from South America and now distributes in nearly all parts of the world [13]. *X. spinosum* was first discovered in Henan province, China in 1981, and then gradually spreads to other provinces such as Liaoning, Xinjiang, Anhui, Gansu, Ningxia, Inner Mongolia in the past decades [14,15,16,17]. The spiny fruits of *X. spinosum* not only cause wool fault problems, as they can contaminate the fleeces of grazing sheep, but is also considered an effective biological characteristic that facilitates this plant’s quick spread [18].

According to the “novel weapons hypothesis”, the exotic *X. spinosum* might release some naive substances into the environment that native species are more sensitive to. It is believed that allelochemicals produced by exotic plants might either directly accumulate in the soil at effective doses to affect neighboring plants’ growth, or by indirectly alter soil properties including community structure of soil microorganisms, physical and chemical characteristics, and so on [19,20]. Our previous investigation revealed that the ethanol extracts of leaves, stems, roots, and fruits of *X. spinosum* all exhibited phytotoxic activity at different levels against receiver plants, with leaf extract being the most potent [21]. Combined with the fact that the leaves represent relatively the largest amount of biomass compared to other plant parts (i.e., stems, roots and fruits) of *X. spinosum*, we selected leaves for further isolation of phytotoxic compounds.

## 2. Results and Discussion

### 2.1. Isolation and Identification of Six Phytotoxic Compounds

**Figure 1:**
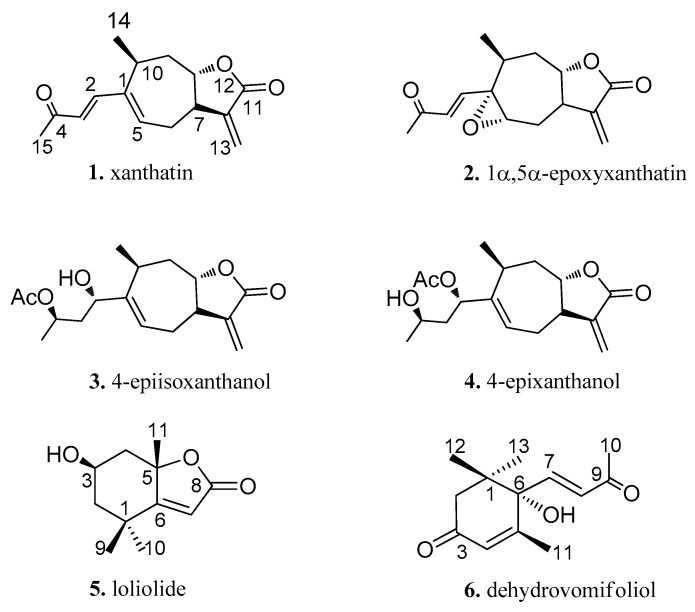
Chemical structures of **1**–**6**.

Column chromatography and semi-preparative HPLC of ethyl acetate extract of leaves of *X. spinosum* led to the isolation of 6 compounds. The compounds were identified as xanthatin (**1**) [22], 1α,5α-epoxyxanthatin (**2**) [23], 4-epiisoxanthanol (**3**) [22], 4-epixanthanol (**4**) [22], loliolide (**5**) [24], and dehydrovomifoliol (**6**) [25], by alignment with spectral data reported in the literature (Figure 1). Of them, compounds 2–6 were isolated from the *X. spinosum* for the first time.

### 2.2. Phytotoxic Effect of Isolated Compounds

**Figure 2:**
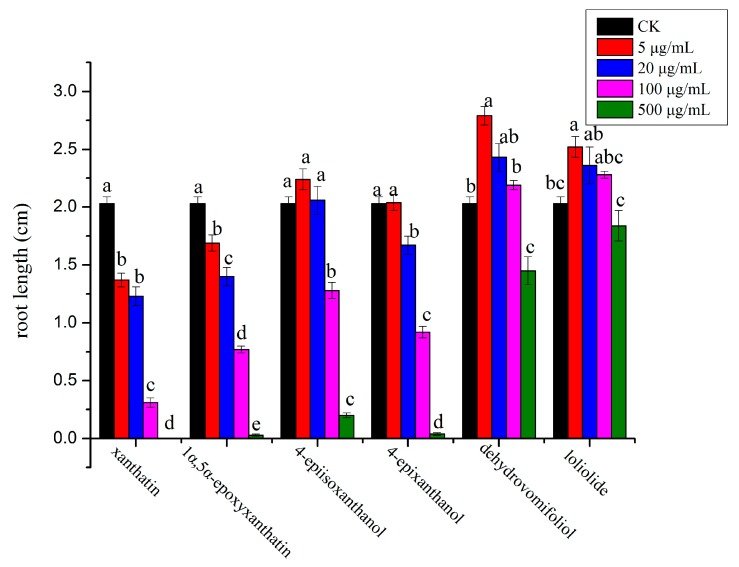
Phytotoxic effect of isolated compounds on root growth of *A. retroflexus*. Different letters represent a significant difference at *p* < 0.05 level according to Fisher’s LSD test.

**Figure 3:**
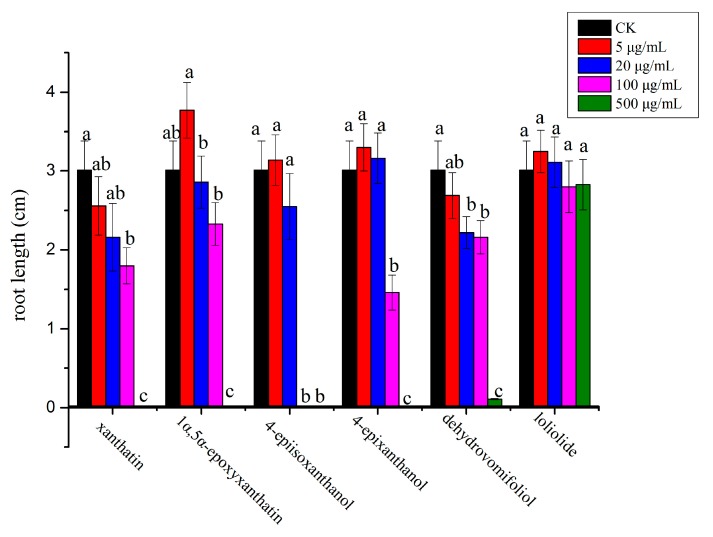
Phytotoxic effect of isolated compounds on root growth of *P. annua*. Different letters represent a significant difference at *p* < 0.05 level according to Fisher’s LSD test.

**Figure 4:**
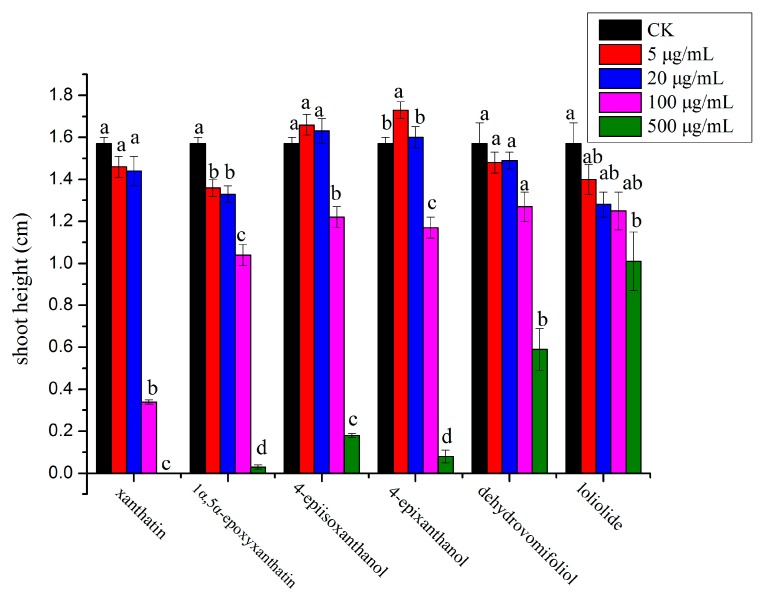
Phytotoxic effect of isolated compounds on shoot growth of *A. retroflexus*. Different letters represent a significant difference at *p* < 0.05 level according to Fisher’s LSD test.

**Figure 5:**
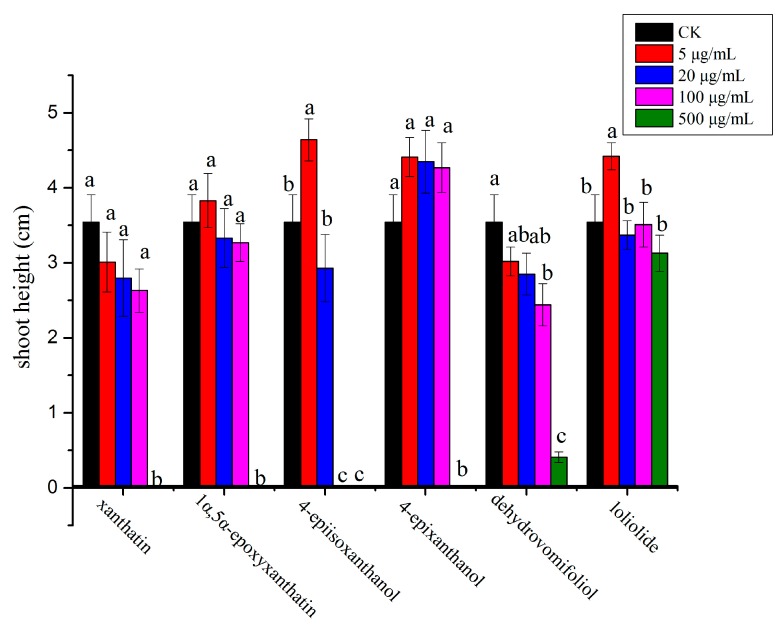
Phytotoxic effect of isolated compounds on shoot growth of *P. annua*. Different letters represent a significant difference at *p* < 0.05 level according to Fisher’s LSD test.

In general, compounds **1**, **3** and **4** exhibited stronger inhibitory effect on the monocot plant, *P. annua,* compared with compound **2**, which showed more potent effect on the dicot plant, *A. retroflexus*. Among the six compounds, compound **1** possessed the strongest inhibitory activity against *A. retroflexus* with an IC_50_ of 28 µg/mL for its root growth; meanwhile, compound **3** showed the most potent activity against *P. annua* with an IC_50_ of 56.8 µg/mL for root growth. Starting from a very low concentration (5 µg/mL), root growth of *A. retroflexus* was significantly inhibited by 32.5% and 16.7% when treated with compounds **1** and **2**, whereas root growth of *A. retroflexus* was significantly increased by 23.9% and 37.2% when treated with compounds **5** and **6**; meanwhile, root growth of *A. retroflexus* was not significantly affected either by compound **3** or **4** at the same concentration. When the concentration was increased to 20 µg/mL, root growth of *A. retroflexus* was significantly inhibited by 39.4%, 31.0% and 17.7%, respectively, when treated with compounds **1**, **2** and **4**; however, it was not significantly affected by compounds **3**, **5** and **6** at such concentration. When the concentration reached 100 µg/mL, root growth of *A. retroflexus* was significantly suppressed by 84.7%, 62.1%, 54.6% and 36.9%, respectively, when compounds **1**–**4** were applied, with compound **1** possessing the strongest activity. At the highest concentration tested (500 µg/mL), seed germination of *A. retroflexus* was mostly completely inhibited by compounds **1**–**4** (Figure 2 and Figure 3). Their phytotoxic effect was similar on shoot growth of receiver plants but at a lesser extent (Figure 4 and Figure 5).

## 3. Discussion

*Xanthium* plants have been used in folk medicine for treating sinusitis, cancers, fever, diabetes, and heart troubles [26,27,28]. In particular, *X. spinosum* was applied against rabies, to relieve chronic fevers, to abate diabetes effect, and even to stimulate saliva production [29,30]. The chemistry of this genus is quite homogeneous: sesquiterpene lactones with guaiane or guaiane frameworks (i.e., xanthanolides) are the main secondary metabolites of most *Xanthium* species [12]. So far, a variety of xanthanolides have been isolated from *Xanthium* plants such as *X. italicum, X. brasilicum*, *X. catharticum*, *X. cavanillesii*, *X. macrocarpum*, *X. orientale*, and so on [12]. Sesquiterpenoid lactones are known for their biological/pharmacological activities. Frequently isolated sesquiterpene lactones such as xanthatin, xanthinol, xanthinosin, xanthinin, etc., have been reported to possess antimicrobial, anti-tumor, anti-leishmanial, anti-ulcerogen, anti-inflammatory, and plant growth regulatory activities [12,22,31,32,33,34]. Among the various biological activities, some xanthanolides such as xanthinin, xanthinosin, xanthatin, and 8-epi-xanthatin have been reported to possess plant growth inhibitory activity; xanthatin seems to be the most potent compound, reducing root growth of ryegrass and Syrian rue by 78% and 96%, respectively, at 50 μg/mL; xanthinin showed weaker activity, 250 μg/mL xanthinin inhibited root growth of lettuce, ryegrass, Syrian rue and redroot pigweed by 33%, and 66%, 94%, 3%, and 99.7%, respectively; as of 8-epi-xanthatin, it was found to be able to inhibit auxin-induced growth of sunflower hypocotyl and oat coleoptile sections at concentrations higher than 100 mM and 30 mM, respectively, and the elongation of cress roots at concentrations higher than 30 mM [9,35,36,37].

Unlike the isolated xanthanolides, compounds **5** and **6** are not limited to *Xanthium* plants and can be found in other species. Both compounds have previously been reported to possess phytotoxic activity [38]. Compound **5** has been isolated from *Marsilea crenata*, *Bunias orientalis*, *Centrostachys aquatic*, etc., and can be found in lots of marine algae with the content ranging from 0.14~4.83 µg/g; and the concentrations required for 50% inhibition of cress and barnyard grass seedlings by loliolide ranged from 32.1 to 128.5 mΜ [39,40,41]. Compound **6** have also been obtained as a phytotoxic substance from *Raphanussativus*, *Chenopodium album*, and *Malva silvestris* [42,43,44]; Interestingly, compounds **5** and **6** have been isolated together as the 2 major allelopathic substances from the herbaceous perennial weed *Paspalum commersonii* [38]. Although the phytotoxicity of compounds **5** and **6** was too weak to be considered as valuable candidates to be further exploited as bioherbicides, the 4 xanthanolides exhibited relatively strong activity, especially compound **1**.

Synthetic herbicides are widely used in weed management, however they are criticized due to their possible involvement of causing serious irreversible environmental problems. Natural herbicides, on the contrary, can be degraded by soil microbes and are thus considered to be environmentally friendly [45]. A number of naturally occurring compounds have been found to possess potent plant inhibitory activity, such as DIMBOA, citral, aspterric acid, cinnacidin, etc., and some of them (or their derivatives/analogues) have been successfully commercialized [46,47,48,49]. For example, mesotrione is a synthesized analogue of leptospermone, which was originally discovered from roots of *Callistemon citrinus* [50]; another herbicide cinmethylin is actually a derivative of 1,4-cineole, which can be found as an important constituent of the essential oils of a number of plants such as *Eucalyptus citriodora* [51,52]; and Endothall is an analogue of the natural compound cantharidin [53]. To the best of our knowledge, this is the first report on the phytotoxicity of 3 xanthanolides, i.e., compounds **2**–**4** and their effective influence on seedling growth of receiver plants implies their potential to be utilized as bioherbicides. 

Besides *X. spinosum*, other *Xanthium* species have also been reported as invasive plants, such as *X. italicum*, *X. strumarium*, *X. mongolicum*, etc., most likely due to their unique biological characteristics [16]. In the case of *X. spinosum*, our results revealed that *X. spinosum* has the ability to produce phytotoxic substances. It is proposed that some exotic plants can affect local ecosystems via allelopathy, by either directly release toxic substances (allelochemicals) into the environment, or indirectly impact aboveground ecosystems by shifting soil physico-chemical properties as well as soil microbial communities [20,54]. Our previous study confirmed the presence of several xanthanolides, i.e., xanthinin, xanthatin, and xanthinosin, with moderate to strong phytotoxicity, in soils infested by another invasive *Xanthium* plant, i.e., *X. italicum*, suggesting these xanthanolides might contribute, at least in part, to the allelopathic property of this plant. However, further work is needed to determine the role of these bioactive compounds in the invasion process of *X. spinosum*, for instance, whether these potential allelochemicals can persist in soil long enough to act as inhibitory agents.

## 4. Conclusions

Six compounds with phytotoxic activities were isolated from the invasive plant *X. spinosum*, and this is the first report of the phytoxicity of compounds **2**–**4**. The compound **3** exhibited significant selective activity between the monocot and dicot receiver species, implying its possible use as a natural selective herbicide. Our work indicated the possible mechanism of *X. spinosum* producing substances with allelopathic potential to suppress growth of native species and to facilitate its invasion success.

## 5. Materials and Methods

### 5.1. Plant Material

Aerial parts of *X. spinosum* were collected in Yining city, Xinjiang province in July 2015. Leaves were separated from the stems and air dried in our laboratory at room temperature for 2 weeks. They were then powdered by an electric grinder for further process.

### 5.2. Extraction and Isolation

Ground leaves of *X. spinosum* (8.6 kg) was macerated in 95% ethanol (8 L) for a week and the plant material was filtered off; the same process was repeated three times. The filtrates were combined and evaporated to yield a residue (90 g) that was fractionated with different solvents on the basis of increasing polarity to afford petroleum ether fraction (72.6 g), ethyl acetate fraction (10 g), and the residue (9.4 g). The ethyl acetate fraction exhibited the strongest phytotoxic activity; therefore it was subjected to column chromatography over a silica gel column eluted with a step gradient elution (PET/EtOAc at 1:0, 5:1, 3:1, 1:1, 1:2, 0:1, and EtOAc/MeOH at 98:2, 96:4, 9:1, 8:2, 0:1). Seventeen fractions were collected based on TLC profiles, and their phytotoxicity was determined at 500 µg/mL against *A. retroflexus*. The most toxic fractions, fractions 4, 5 and 7, were selected for further purification. Fraction 4 was recrystallized in methanol to yield compound **1** (20.3 mg). Fraction 5 was subjected to HPLC with a Phenomenex C18 Column (10 μm, 250 × 10 mm) and methanol/water (3:7–7:3) as the eluent at a flow rate of 3 mL/min. This process afforded the following products: compound **2** (12.7 mg), compound **3** (36.9 mg), compound **5** (4.0 mg) and compound **6** (4.7 mg). Fraction 7 was chromatographed by HPLC with a Phenomenex C18 Column (10 μm, 250 × 10 mm) and methanol/water (3:7–7:3) as the at a flow rate of 3 mL/min to give compound **4** (5.1 mg). To determine the chemical structures of the purified compounds with phytotoxic activity, their spectral data were measured as under: ^1^H and ^13^C NMR spectra were recorded on a Varian Inova-400 instrument (Varian, Boulder, CO, USA) with TMS as an internal standard and CD_3_OD as solvent, and EIMS was performed using a 4000 QTRAP Elite LC-MS/MS system from Applied Biosystems/MDS Sciex (Concord, ON, Canada) coupled with an electrospray ionisation (ESI).

### 5.3. Compounds Characterization

Compound **1**: was obtained as a pale yellowish oil, and its molecular formula was deduced to be C_15_H_18_O_3_ from the ESI-MS (*m*/*z* 247.7 [M + H]^+^), as was consistent with mass spectral data of xanthatin reported in the literature (see Figure S3) [22]; ^1^H NMR (400 MHz, CDCl_3_, *δ* ppm): 2.29 (*s*, 3H, 15-CH_3_), 1.14 (*d*, 3H, *J* = 8.0 Hz, 14-CH_3_), 7.06 (*d*, 1H, *J* = 16.0 Hz, 2-H), 6.27 (*dd*, 1H, *J* = 4.0, 8.0 Hz, 5-H), 6.20 (*d*, 1H, *J* = 16.0, 3-H), 6.19 (*d*, 1H, *J* = 4.0, 13-H), 5.47 (*d*, 1H, *J* = 4.0, 13′-H), 4.27 (*t*, 1H, *J* = 12.0, 8-H), 3.05 (*m*, 1H, 7-H), 2.79 (*m*, 1H, 6β-H), 2.55 (*m*, 1H, 10-H), 2.38 (*td*, 1H, *J* = 4.0, 12.0 Hz, 9β-H), 2.22 (*td*, 1H, *J* = 4.0, 12.0 Hz, 9α-H), 1.84 (*td*, *J* = 4.0, 16.0 Hz, 1H, 6α-H); ^13^C NMR (100 MHz, CDCl_3_, *δ* ppm): 18.83 (14-C), 27.21 (6-C), 27.88 (15-C), 29.16 (10-C), 36.60 (9-C), 47.46 (7-C), 81.47 (8-C), 118.91 (13-C), 124.67 (3-C), 137.97 (5-C), 139.22 (11-C), 144.78 (1-C), 148.44 (2-C), 169.62 (12-C), 198.43 (4-C). They were also identical to the data of xanthatin reported in the literature [22]. From these spectral data, the structure of **1** was elucidated as xanthatin.

Compound **2**: was obtained as a pale yellowish oil, and its molecular formula was deduced to be C_15_H_18_O_4_ from the ESI-MS (*m*/*z* 263.8 [M + H]^+^), as was consistent with mass spectral data of 1α,5α-epoxyxanthatin reported in the literature [23]; ^1^H NMR (400 MHz, CDCl_3_, *δ* ppm): 2.24 (*s*, 3H, 15-CH_3_), 1.27 (*d*, 3H, *J* = 8.0 Hz, 14-CH_3_), 6.90 (*d*, 1H, *J* = 16.0 Hz, 2-H), 6.28 (*d*, 1H, *J* = 16.0, 3-H), 6.15 (*d*, 1H, *J* = 4.0, 13-H), 5.44 (*d*, 1H, *J* = 4.0, 13′-H), 3.91 (*td*, 1H, *J* =4.0, 12.0, 8-H), 3.05 (*d*, 1H, *J* = 4.0, 5-H), 2.88 (*m*, 1H, 7-H), 2.65 (*m*, 1H, 6β-H), 2.59 (*m*, 1H, 10-H), 2.11 (*dt*, 1H, *J* = 4.0, 12.0 Hz, 9β-H), 2.0 (*dt*, 1H, *J* = 4.0, 12.0 Hz, 9α-H), 1.90 (*d*, *J* = 4.0, 1H, 6α-H); ^13^C NMR (100 MHz, CDCl_3_, *δ* ppm): 16.34 (14-C), 26.27 (15-C), 28.22 (9-C), 30.47 (6-C), 34.41 (10-C), 42.85 (7-C), 63.59 (5-C), 64.93 (1-C), 80.14 (8-C), 118.53 (13-C), 129.03 (3-C), 139.43 (11-C), 145.03 (2-C), 169.37 (12-C), 197.90 (4-C). They were also identical to the data of 1α,5α-epoxyxanthatin reported in the literature [23]. From these spectral data, the structure of **2** was elucidated as 1α,5α-epoxyxanthatin.

Compound **3**: was obtained as a yellowish oil, and its molecular formula was deduced to be C_17_H_24_O_5_ from the ESI-MS (*m*/*z* 307.4 [M − H]^−^), as was consistent with mass spectral data of 4-epiisoxanthanol reported in the literature [22]; ^1^H NMR (400 MHz, CD_3_OD, *δ* ppm): 2.01 (*d*, 3H, *J* = 4.0 Hz, OAC-CH_3_), 1.21 (*d*, 3H, *J* = 8.0 Hz, 15-CH_3,_), 1.19 (*d*, 3H, *J* = 8.0 Hz, 14-CH_3_), 6.07 (*d*, 1H, *J* = 4.0, 13-H), 5.71 (*br*, *dd*, 1H, *J* = 4.0,12.0, 5-H), 5.54 (*d*, 1H, *J* = 4.0, 13′-H), 4.37 (*td*, 1H, *J* = 4.0, 12.0, 4-H), 4.08 (*t*, 1H, *J* = 8.0, 8-H), 3.29 (*t*, 1H, *J* =4.0, 2-H), 2.79 (*m*, 1H, 10-H), 2.54 (*ddd*, 1H, *J* = 4.0, 8.0 Hz, 6α-H), 2.47 (*ddd*, 1H, *J* = 4.0, 8.0 Hz, 7-H), 2.28 (*ddd*, 1H, *J* = 4.0, 8.0 Hz, 9β-H), 2.12 (*ddd*, 1H, *J* = 4.0, 8.0 Hz, 6β-H), 1.91 (*m*, 1H, 9α-H), 1.66 (*m*, 2H, 3, 3′-H); ^13^C NMR (100 MHz, CD_3_OD, *δ* ppm): 18.82 (14-C), 19.06 (15-C), 19.84 (OAC-CH_3_), 24.71 (6-C), 28.52 (10-C), 36.49 (9-C), 40.54 (3-C), 48.25 (7-C), 68.70 (4-C), 75.80 (2-C), 82.77 (8-C), 117.58 (13-C), 124.50 (5-C), 140.00 (11-C), 148.75 (1-C), 170.63 (12-C), 171.04 (OAC-C). They were also identical to the data of 4-epiisoxanthanol reported in the literature [22]. From these spectral data, the structure of **3** was elucidated as 4-epiisoxanthanol.

Compound **4**: was obtained as a yellowish oil, and its molecular formula was deduced to be C_17_H_24_O_5_ from the ESI-MS (*m*/*z* 307.4 [M − H]^−^), as was consistent with mass spectral data of 4-epixanthanol reported in the literature [22]; ^1^H NMR (400 MHz, CD_3_OD, *δ* ppm): 2.00 (*d*, 3H, *J* = 4.0 Hz, OAC-CH_3_), 1.21 (*t*, 3H, *J* = 8.0 Hz, 15-CH_3_), 1.14 (*d*, 3H, *J* = 8.0 Hz, 14-CH_3,_), 6.07 (*d*, 1H, *J* = 4.0, 13-H), 5.97 (*dd*, 1H, *J* = 4.0,12.0, 5-H), 5.54 (*d*, 1H, *J* = 4.0, 13′-H), 5.32 (*t*, 1H, *J* = 8.0, 2-H), 4.37 (*td*, 1H, *J* =4.0, 12.0, 8-H), 3.69 (*m*, 1H, 4-H), 2.79 (*m*, 1H, 10-H), 2.62 (*ddd*, 1H, *J* = 4.0, 8.0 Hz, 6α-H), 2.47 (*ddd*, 1H, *J* = 4.0, 8.0 Hz, 7-H), 2.28 (*ddd*, 1H, *J* = 4.0, 8.0 Hz, 9β-H), 2.14 (*ddd*, 1H, *J* = 4.0, 8.0 Hz, 6β-H), 1.89 (*m*, 1H, 9α-H), 1.66 (*m*, 2H, 3, 3′-H); ^13^C NMR (100 MHz, CD_3_OD, *δ* ppm): 18.37 (14-C), 19.80 (OAC-CH_3_), 22.38 (15-C), 24.64 (6-C), 29.11 (10-C), 36.54 (9-C), 41.54 (3-C), 48.10 (7-C), 64.13 (4-C), 78.76 (2-C), 82.49 (8-C), 117.59 (13-C), 127.45 (5-C), 139.85 (11-C), 144.75 (1-C), 170.50 (12-C), 171.06 (OAC-C). They were also identical to the data of 4-epixanthanol reported in the literature [22]. From these spectral data, the structure of **4** was elucidated as 4-epixanthanol.

Compound **5**: was obtained as a colorless crystal, and its molecular formula was deduced to be C_11_H_16_O_3_ from the ESI-MS (*m*/*z* 197.5 [M + H]^+^), as was consistent with mass spectral data of loliolide reported in the literature [24]; ^1^H NMR (400 MHz, CD_3_OD, *δ* ppm): 1.74 (*s*, 3H, 11-CH_3_), 1.45 (*s*, 3H, 9-CH_3_), 1.26 (*s*, 3H, 10-CH_3_), 4.19 (*s*, 1H, 3-H), 2.46 (*dt*, 1H, *J* = 4.0, 16.0, 4β-H), 1.98 (*dt*, 1H, *J* = 4.0, 16.0, 2β-H), 1.71 (*d*, 1H, *J* = 4.0, 4α-H), 1.50 (*ddd*, 1H, *J* = 4.0, 8.0 Hz, 2α-H); ^13^C NMR (100 MHz, CD_3_OD, *δ* ppm): 25.52 (9-CH_3_), 25.99 (11-CH_3_), 29.57 (10-CH_3_), 35.75 (1-C), 44.99 (4-C), 46.53 (2-C), 65.80 (3-C), 87.52 (5-C), 111.88 (7-C), 172.12 (6-C), 184.13 (8-C). They were also identical to the data of loliolide reported in the literature [25]. From these spectral data, the structure of **5** was elucidated as loliolide.

Compound **6**: was obtained as a white crystal, and its molecular formula was deduced to be C_13_H_18_O_3_ from the ESI-MS (*m*/*z* 221.3 [M − H]^+^), as was consistent with mass spectral data of dehydrovomifoliol reported in the literature [25]; ^1^H NMR (400 MHz, CD_3_OD, *δ* ppm): 2.29 (*s*, 3H, 10-CH_3_), 1.87 (*d*, 3H, *J* = 8.0 Hz, 11-CH_3_), 1.05 (*s*, 3H, 13-CH_3,_), 1.00 (*s*, 3H, 12-CH_3,_), 6.98 (*d*, 1H, *J* = 16.0, 7-H), 6.42 (*d*, 1H, *J* =16.0, 8-H), 5.92 (*s*, 1H, 4-H), 2.56 (*s*, 1H, 2α-H), 2.24 (*s*, 1H, 2β-H); ^13^C NMR (100 MHz, CD_3_OD, *δ* ppm): 17.71 (11-C), 22.07 (12-CH_3_), 23.30 (13-CH_3_), 26.19 (10-CH_3_), 41.21 (1-C), 49.08 (2-C), 78.59 (6-C), 126.59 (4-C), 130.27 (8-C), 146.90 (7-C), 163.20 (5-C), 198.92 (3-C), 199.21 (9-C). They were also identical to the data of dehydrovomifoliol reported in the literature [25]. From these spectral data, the structure of **6** was elucidated as dehydrovomifoliol.

### 5.4. Phytotoxicity Bioassays

A dicot plant, *Amaranthus retroflexus* L., and a monocot plant, *Poa annua* L., were used as receiver plants for testing the phytotoxic activity of the isolated compounds. Seeds of *A. retroflexus* and *P. annua* were surface sterilized with 0.5% HgCl_2_ before use. Isolated compounds were dissolved in methanol to give solutions at 5, 20, 100, 500 μg/mL and added to petri dishes (3 cm diameter) lined with filter papers. After complete evaporation of methanol, distilled water (1 mL) was added to each petri dish followed by addition of 10 seeds. All petri dishes were stored in the dark at 25 °C. Seedlings were allowed to grow for 6 (for *A. retroflexus*) or 8 (for *P. annua*) days before shoot and root length were measured. Three replicates were made for each treatment (N = 30).

### 5.5. Statistical Analyses

The significance of the phytotoxic activity of compounds **1**–**6** on seedling growth of receiver species was first examined by ANOVA (*p* < 0.05) and then analyzed using Fisher’s LSD (Least Significant Difference) test at *p* < 0.05 level. All statistical analyses were performed with SPSS 13.

## Supplementary Materials

The following are available online. Figure S1: ^1^H NMR spectrum of xanthatin (**1**) (CDCl_3_, 400 MHz), Figure S2: ^13^C NMR spectrum of xanthatin (**1**) (CDCl_3_, 100 MHz), Figure S3: Mass spectrum of xanthatin (**1**), Figure S4: ^1^H NMR spectrum of 1α,5α-epoxyxanthatin (**2**) (CDCl_3_, 400 MHz), Figure S5: ^13^C NMR spectrum of 1α,5α-epoxyxanthatin (**2**) (CDCl_3_, 100 MHz), Figure S6: Mass spectrum of 1α,5α-epoxyxanthatin (**2**), Figure S7: ^1^H NMR spectrum of 4-epiisoxanthanol (**3**) (CD_3_OD, 400 MHz), Figure S8: ^13^C NMR spectrum of 4-epiisoxanthanol (**3**) (CD_3_OD, 100 MHz), Figure S9: Mass spectrum of 4-epiisoxanthanol (**3**), Figure S10: ^1^H NMR spectrum of 4-epiisoxanthanol (**4**) (CD_3_OD, 400 MHz), Figure S11: ^13^C NMR spectrum of 4-epiisoxanthanol (**4**) (CD_3_OD, 100 MHz), Figure S12: Mass spectrum of 4-epiisoxanthanol (**4**), Figure S13: ^1^H NMR spectrum of loliolide (**5**) (CD_3_OD, 400 MHz), Figure S14: ^13^C NMR spectrum of loliolide (**5**) (CD_3_OD, 100 MHz), Figure S15: Mass spectrum of loliolide (**5**), Figure S16: ^1^H NMR spectrum of dehydrovomifoliol (**6**) (CD_3_OD, 400 MHz), Figure S17: ^13^C NMR spectrum of dehydrovomifoliol (**6**) (CD_3_OD, 100 MHz), Figure S18: Mass spectrum of dehydrovomifoliol (**6**).
