# Supplementary material for: Phytotoxic Compounds Isolated from Leaves of the Invasive Weed Xanthium spinosum
Source: Molecules. 2018 Nov 1;23(11):2840. doi: 10.3390/molecules23112840 (PMC6278460; doi:10.3390/molecules23112840)
Supplement: Supplementary file 1 [file molecules-23-02840-s001.pdf]

SUPPLEMENTARY MATERIAL

Phytotoxic Compounds Isolated from Leaves of the Invasive Weed *Xanthium Spinosum*

Zhuogeng Yuan [1], Xiangwei Zheng[2], Yu Zhao[1], Ying Liu[1], Shixing Zhou[3], Caixia Wei[3], Yunxia Hu[1,*] and Hua Shao[3,*]

[1] Yili Normal University, Yining, 835000, China; Emails: yzg821871139@outlook.com (Z.Y.); 2001 zhaoyu@sohu.com(Y.Z.); zylyzhlily@126.com(Y.L.).

[2] Engineering Research Center of Modern Preparation Technology of TCM, Ministry of Education, Shanghai University of Traditional Chinese Medicine, Shanghai,201203, China; Email: zhengxwsh@hotmail.com (X.Z.);

[3] Key Laboratory of Biogeography and Bioresource in Arid Land, Xinjiang Institute of Ecology andGeography, Chinese Academy of Sciences, Urumqi 830011, China; E-Mails:zhoushixing16@mails.ucas.ac.cn (S.Z.); weicaixia16@mails.ucas.ac.cn (C.W.)

* Correspondence: yunxiahu1214@163.com (Y.H.);shaohua@ms.xjb.ac.cn (H.S.); Tel.: +86-991-7823155

List of content

Figure S1. ^{1}H NMR spectrum of xanthatin (**1**) ($CDCl_3$, 400 MHz).
Figure S2. ^{13}C NMR spectrum of xanthatin (**1**) ($CDCl_3$, 100 MHz).
Figure S3. Mass spectrum of xanthatin (**1**).
Figure S4. ^{1}H NMR spectrum of 1α, 5α-epoxyxanthatin (**2**) ($CDCl_3$, 400 MHz).
Figure S5. ^{13}C NMR spectrum of 1α, 5α-epoxyxanthatin (**2**) ($CDCl_3$, 100 MHz).
Figure S6. Mass spectrum of 1α, 5α-epoxyxanthatin (**2**).
Figure S7. ^{1}H NMR spectrum of 4-epiisoxanthanol (**3**) (CD_3OD, 400 MHz).
Figure S8. ^{13}C NMR spectrum of 4-epiisoxanthanol (**3**) (CD_3OD, 100 MHz).
Figure S9. Mass spectrum of 4-epiisoxanthanol (**3**).
Figure S10. ^{1}H NMR spectrum of 4-epixanthanol (**4**) (CD_3OD, 400 MHz).
Figure S11. ^{13}C NMR spectrum of 4-epixanthanol (**4**) (CD_3OD, 100 MHz).
Figure S12. Mass spectrum of 4-epixanthanol (**4**).
Figure S13. ^{1}H NMR spectrum of loliolide (**5**) (CD_3OD, 400 MHz).
Figure S14. ^{13}C NMR spectrum of loliolide (**5**) (CD_3OD, 100 MHz).
Figure S15. Mass spectrum of loliolide (**5**).
Figure S16. ^{1}H NMR spectrum of dehydrovomifoliol (**6**) (CD_3OD, 400 MHz).
Figure S17. ^{13}C NMR spectrum of dehydrovomifoliol (**6**) (CD_3OD, 100 MHz).
Figure S18. Mass spectrum of dehydrovomifoliol (**6**) .

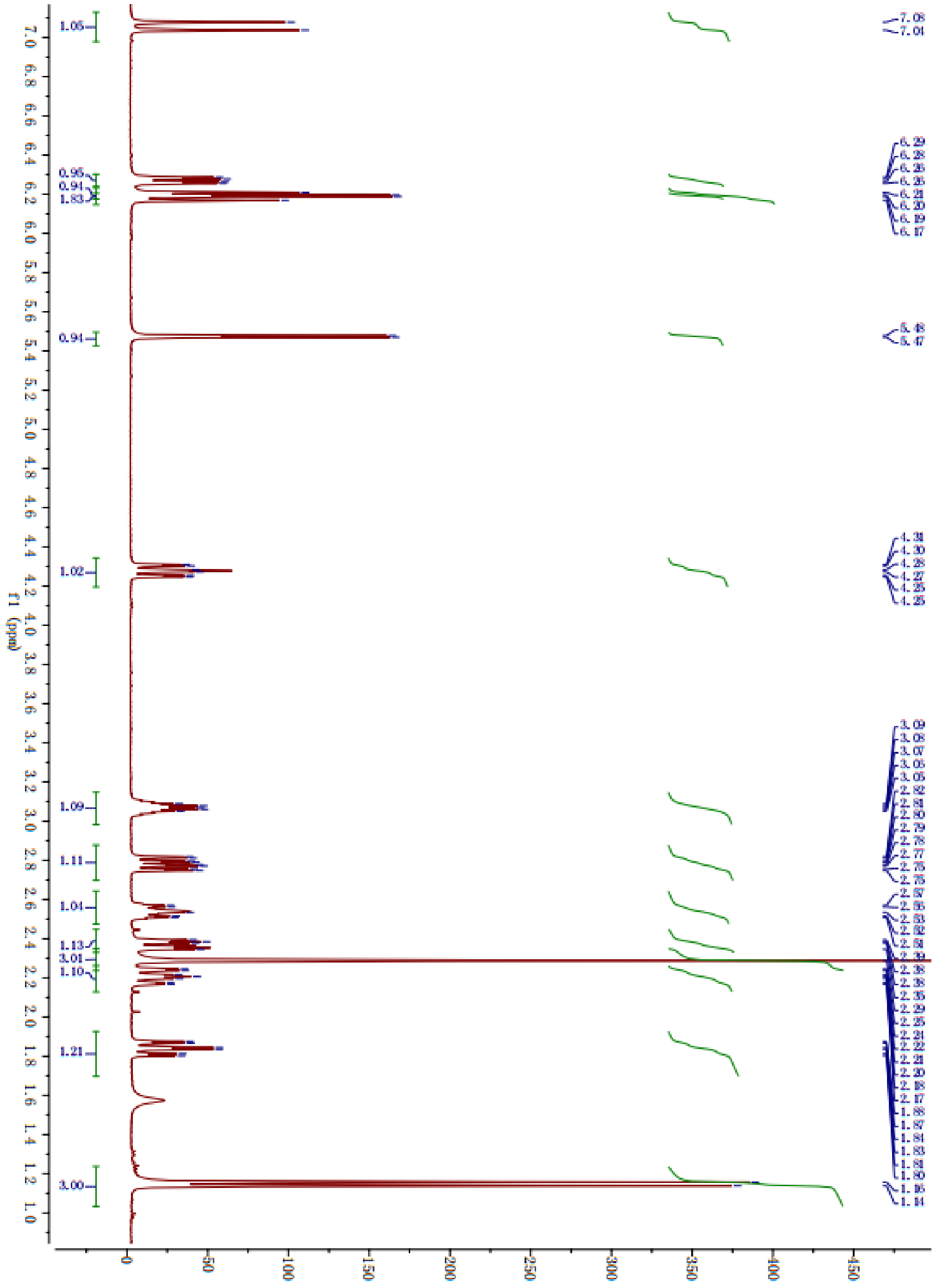

Figure S1. ^{1}H NMR spectrum of xanthatin (**1**) ($CDCl_3$, 400 MHz).

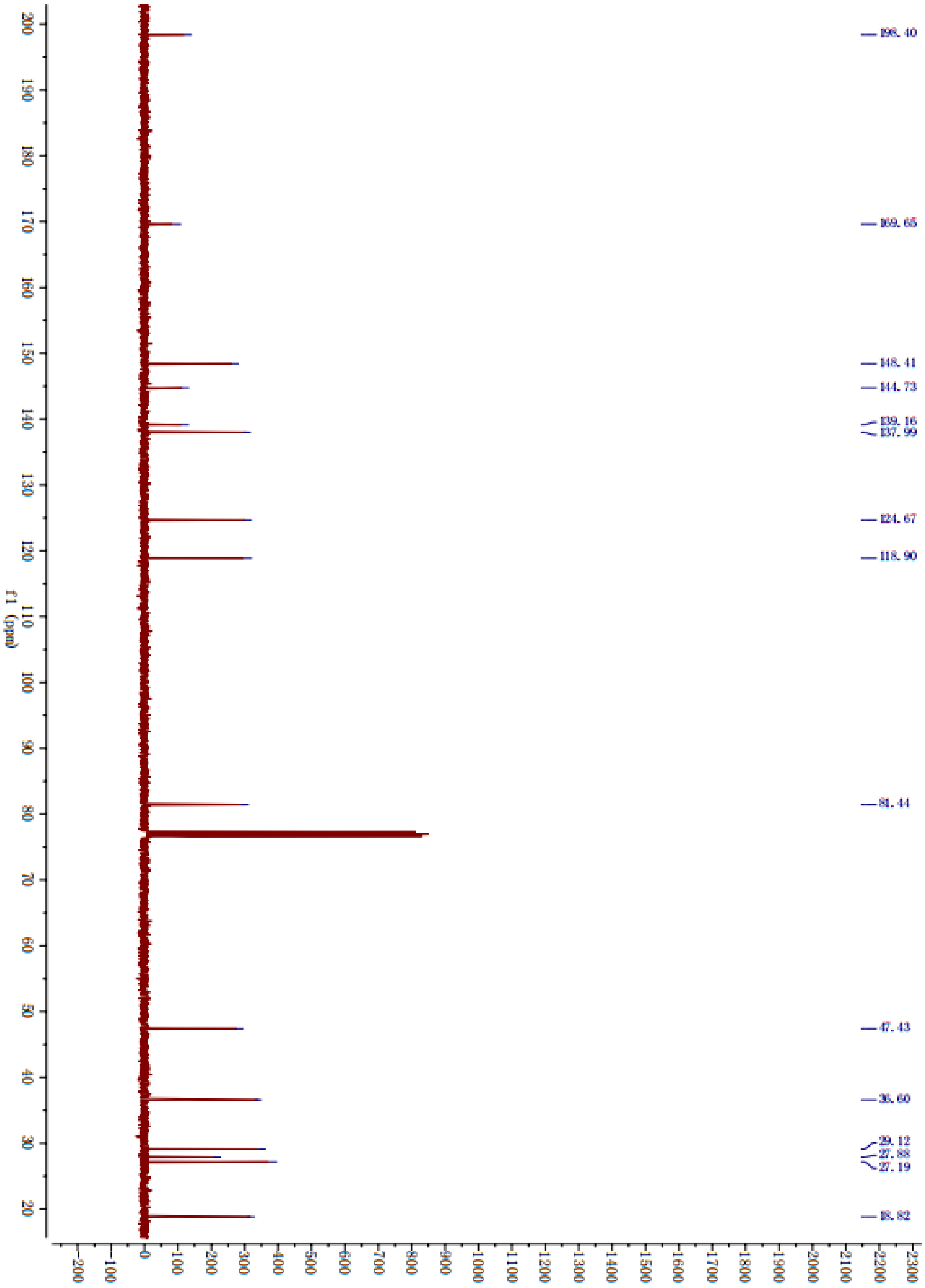

Figure S2. ^{13}C NMR spectrum of xanthatin (**1**) ($CDCl_3$, 100 MHz).

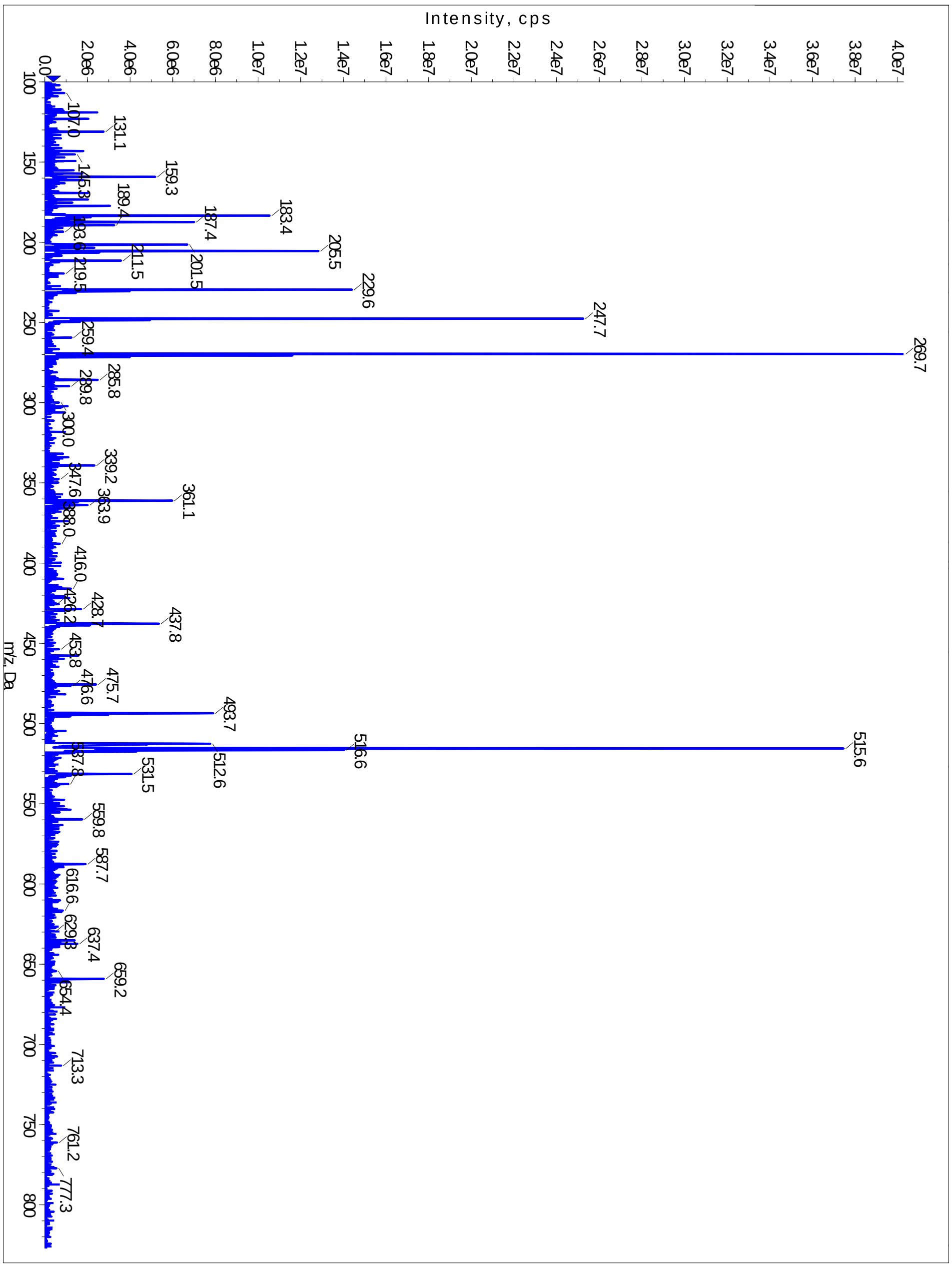

Figure S3. Mass spectrum of xanthatin (**1**).

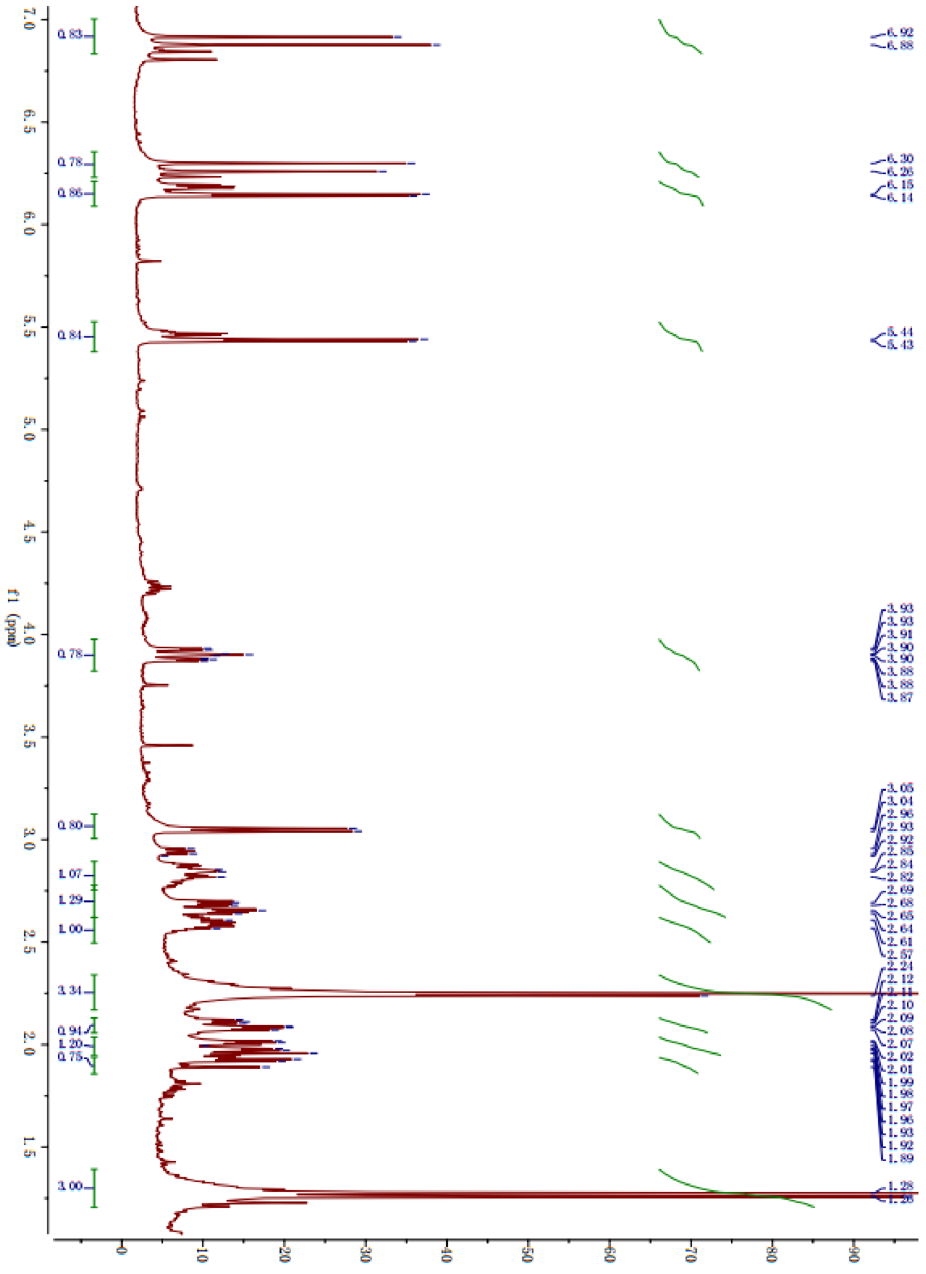

Figure S4. ^{1}H NMR spectrum of 1α, 5α-epoxyxanthatin (**2**) ($CDCl_3$, 400 MHz).

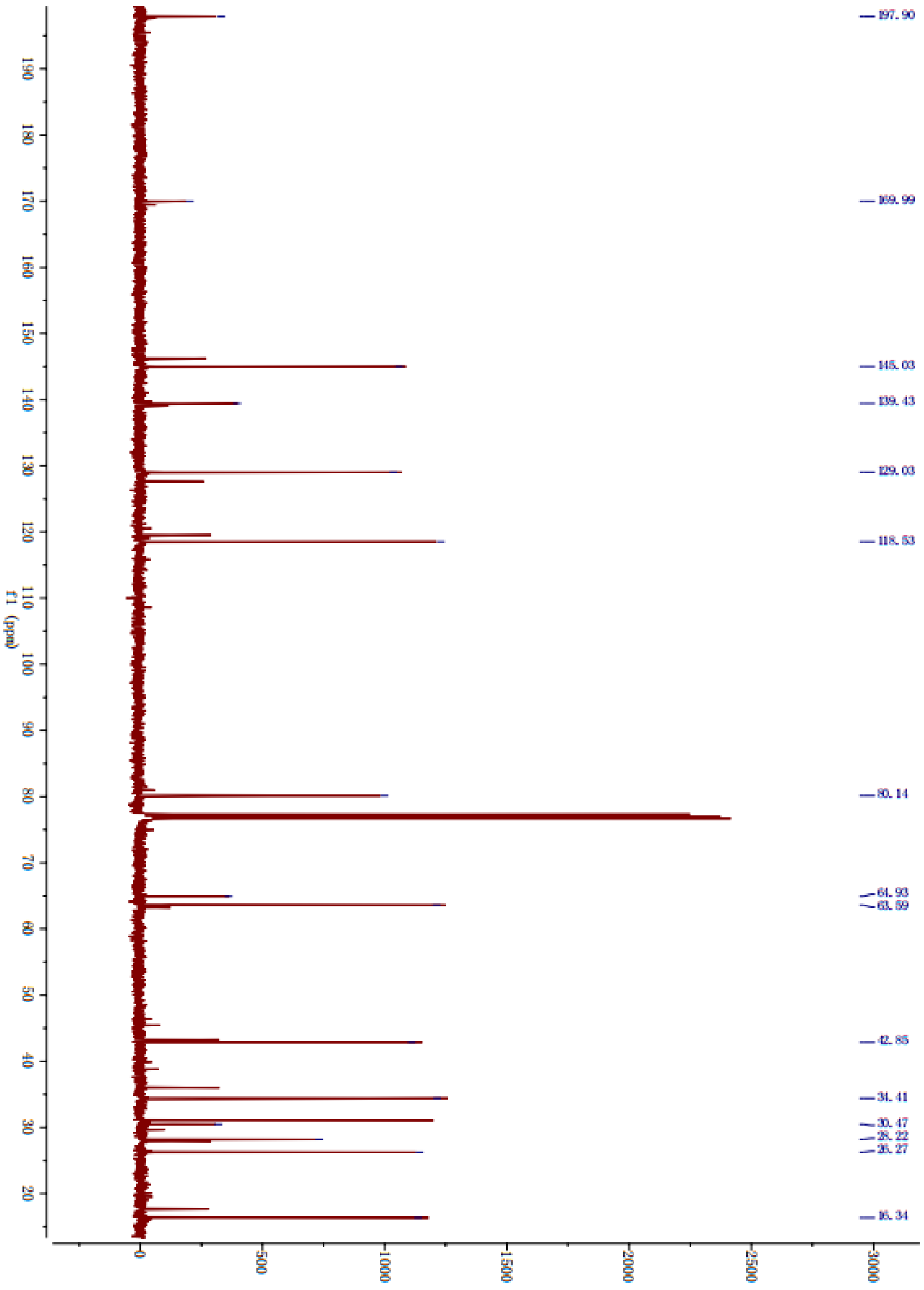

Figure S5. ^{13}C NMR spectrum of 1α, 5α-epoxyxanthatin (**2**) ($CDCl_3$, 100 MHz).

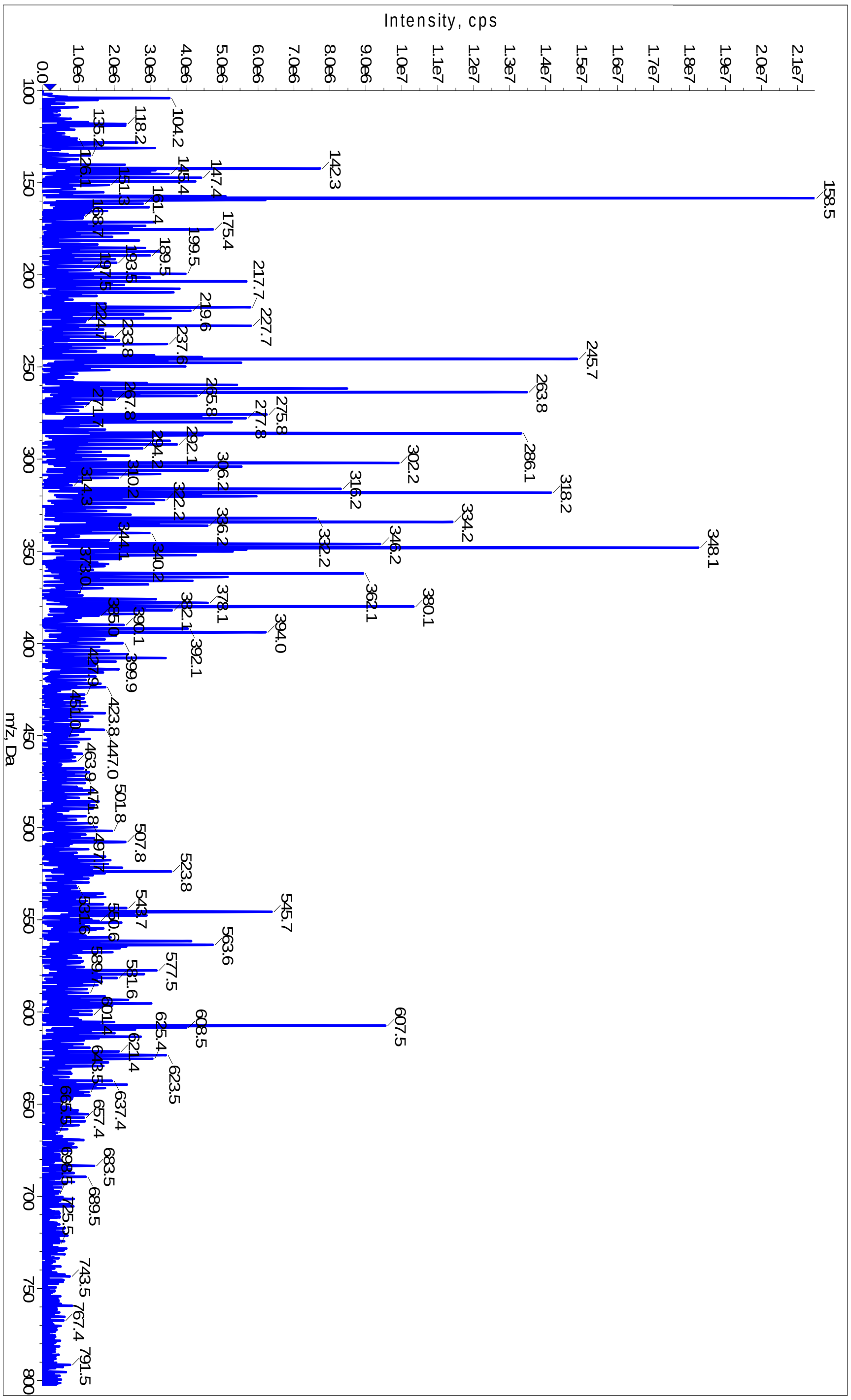

Figure S6. Mass spectrum of 1α, 5α-epoxyxanthatin (**2**).

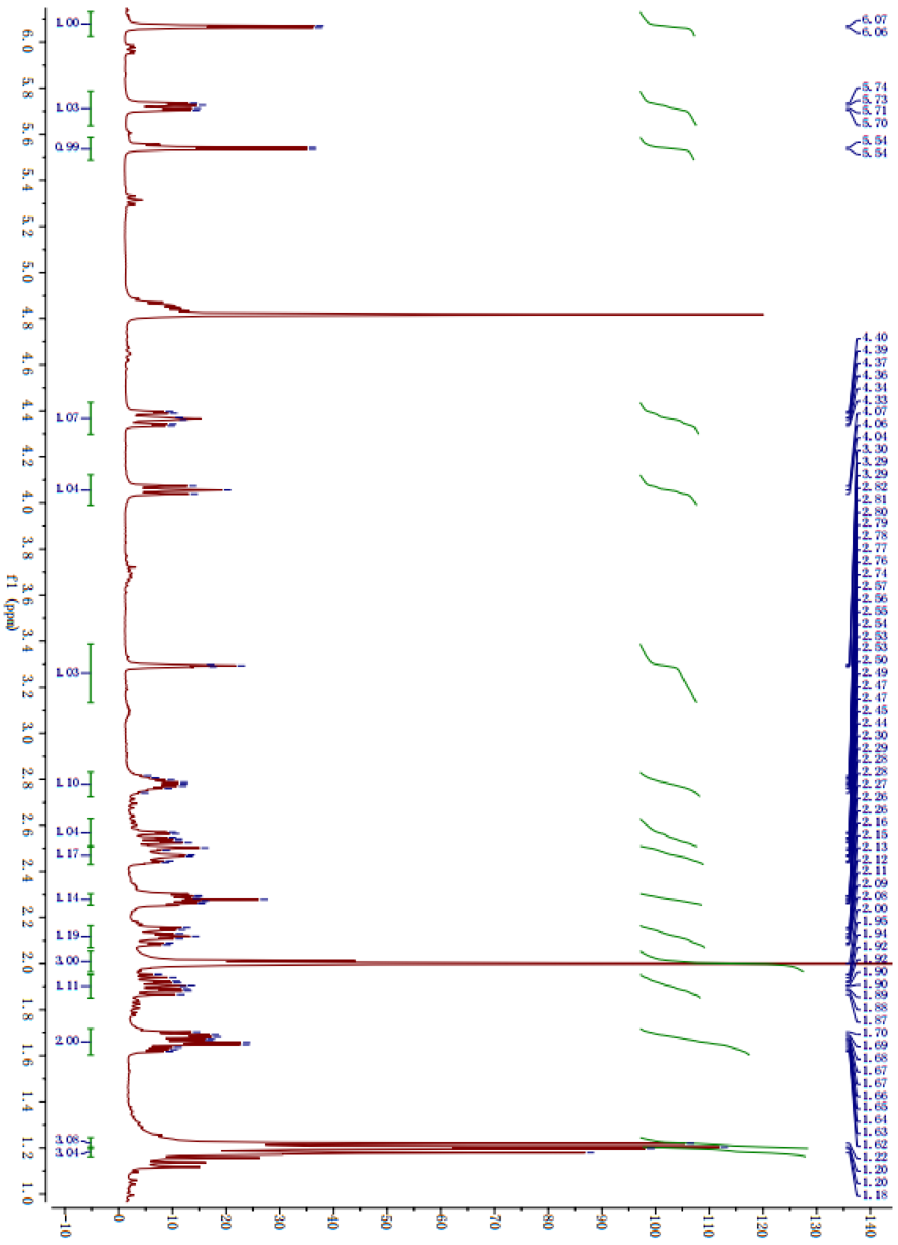

Figure S7. ^{1}H NMR spectrum of 4-epiisoxanthanol (**3**) (CD_3OD, 400 MHz).

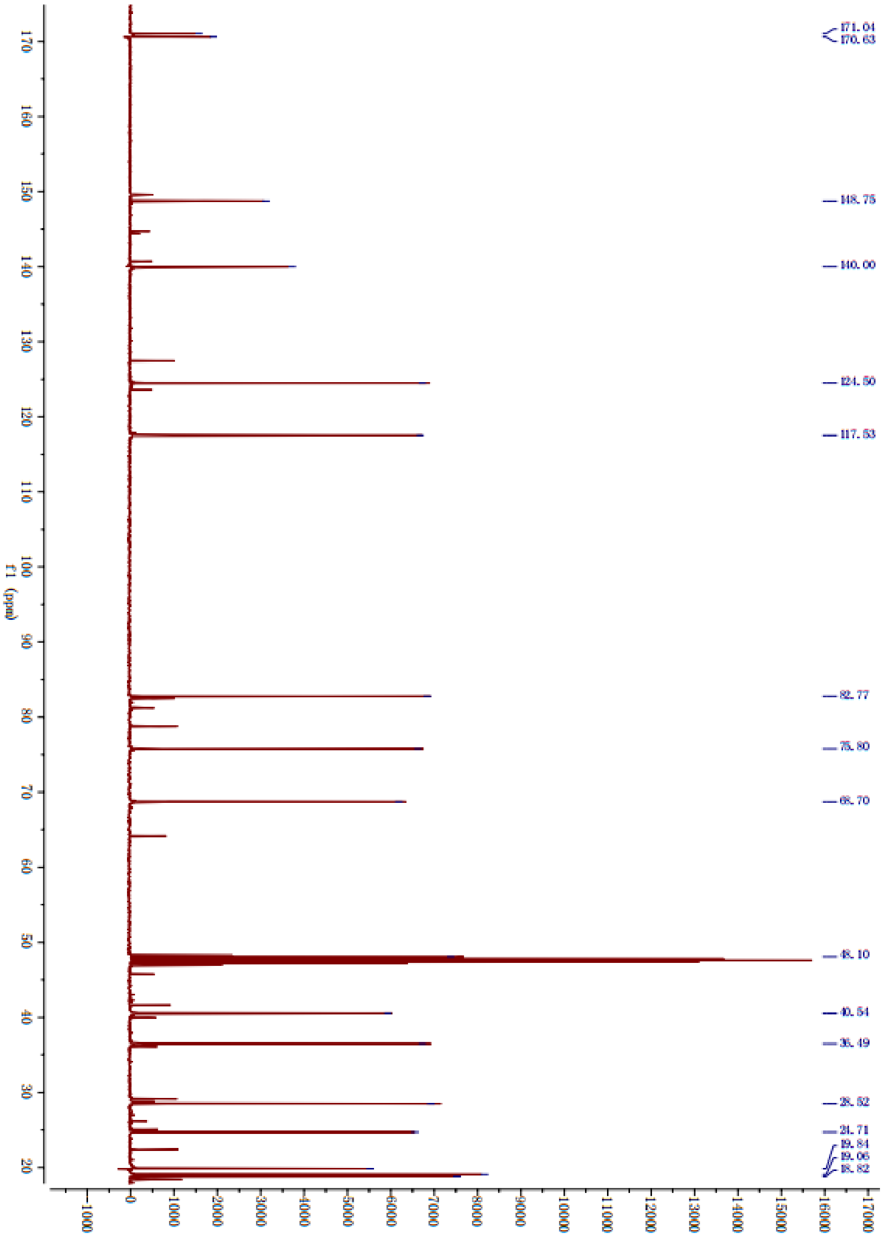

Figure S8. ^{13}C NMR spectrum of 4-epiisoxanthanol (**3**) (CD_3OD, 100 MHz).

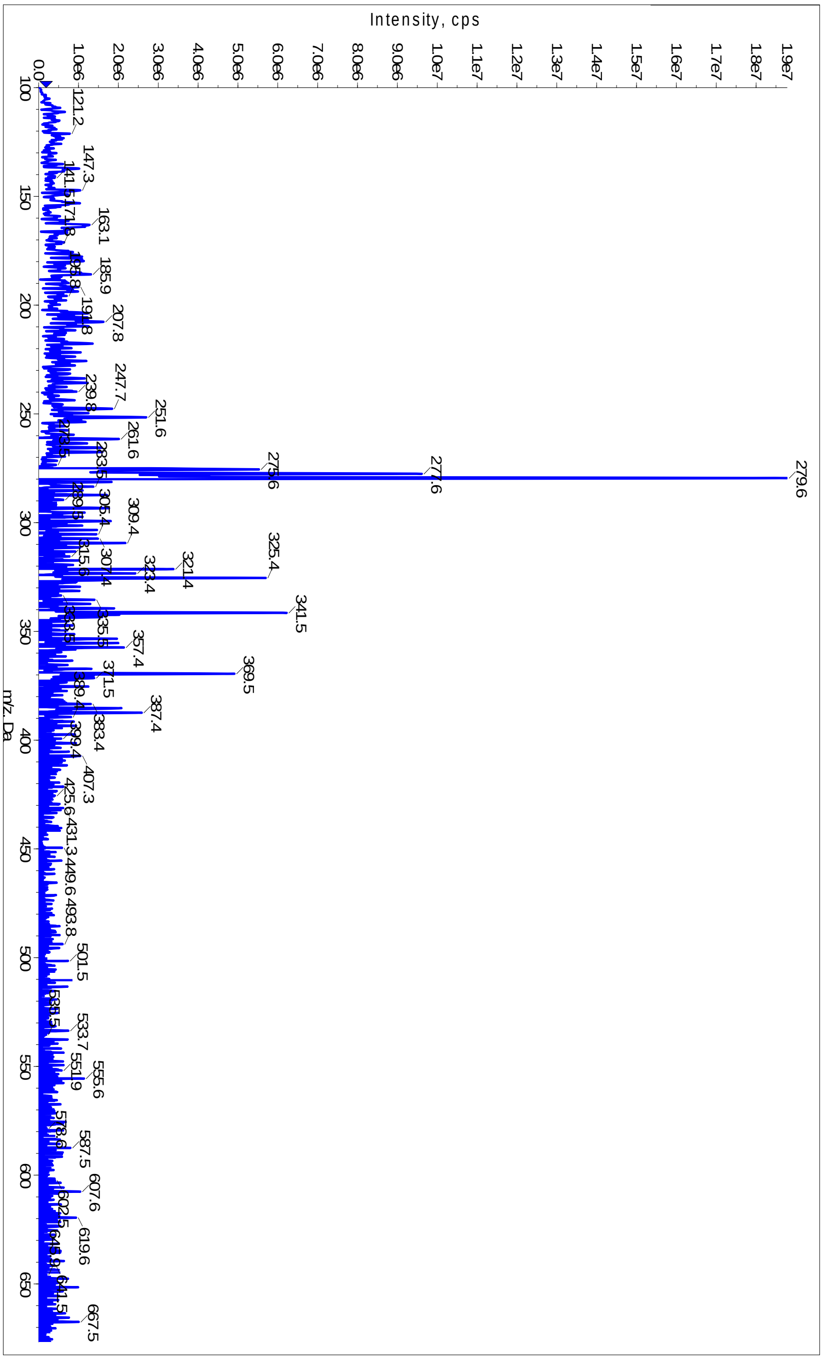

Figure S9. Mass spectrum of 4-epiisoxanthanol (**3**).

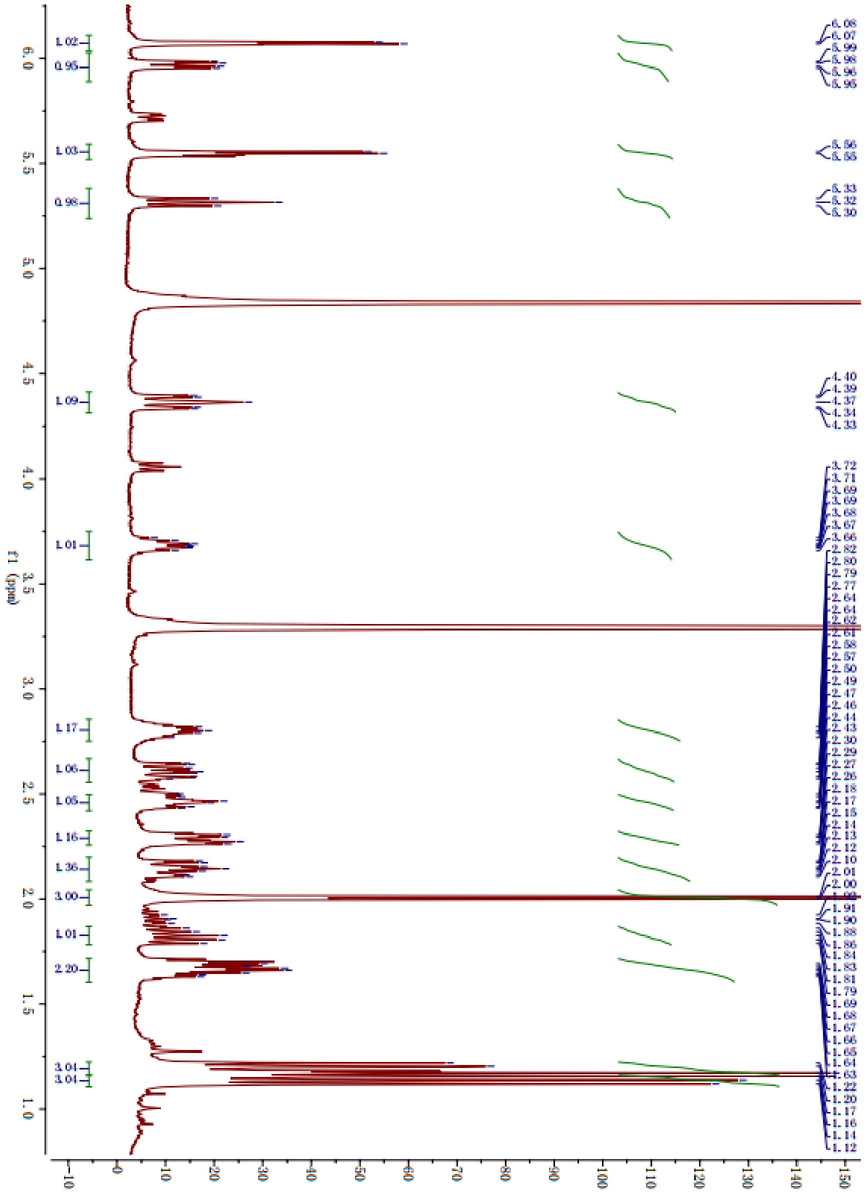

Figure S10. ^{1}H NMR spectrum of 4-epixanthanol (**4**) (CD_3OD, 400 MHz).

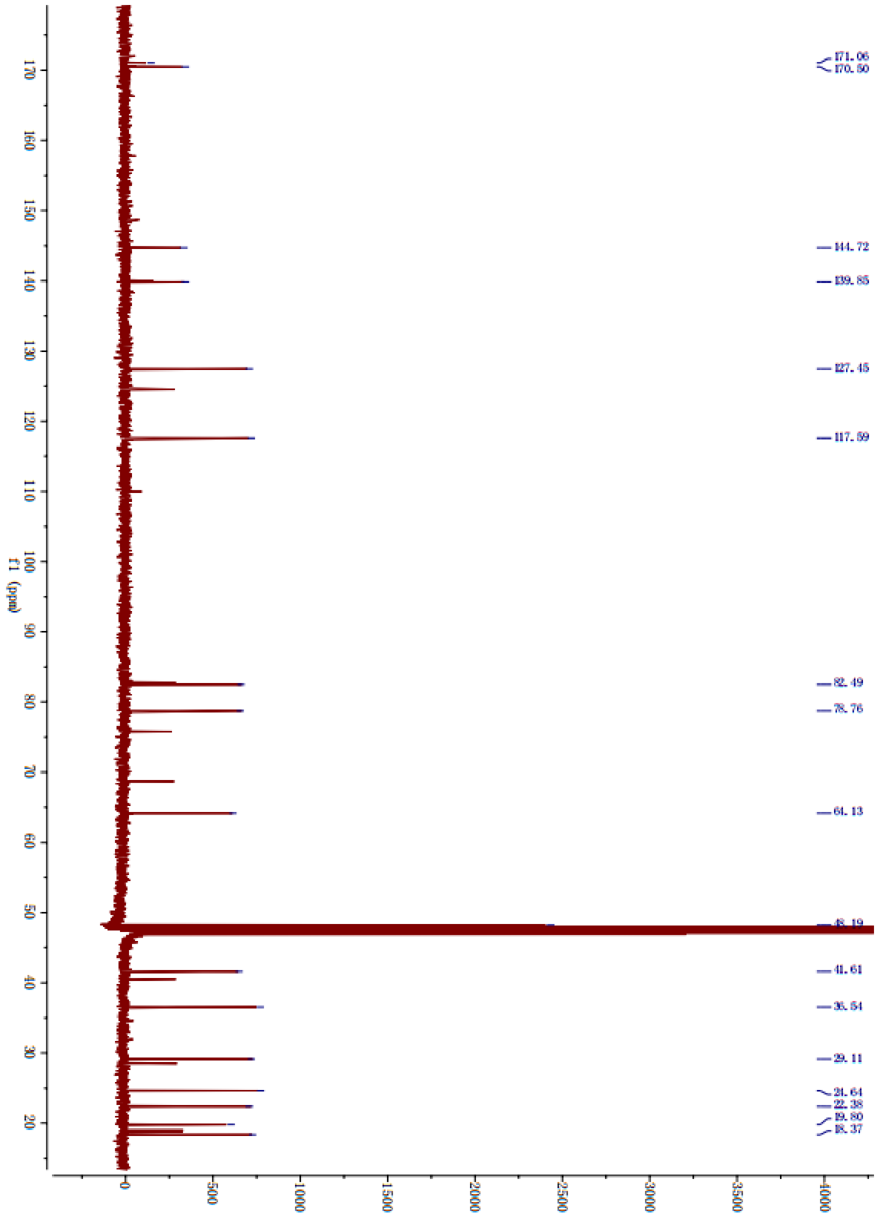

Figure S11. ^{13}C NMR spectrum of 4-epixanthanol (**4**) (CD_3OD, 100 MHz).

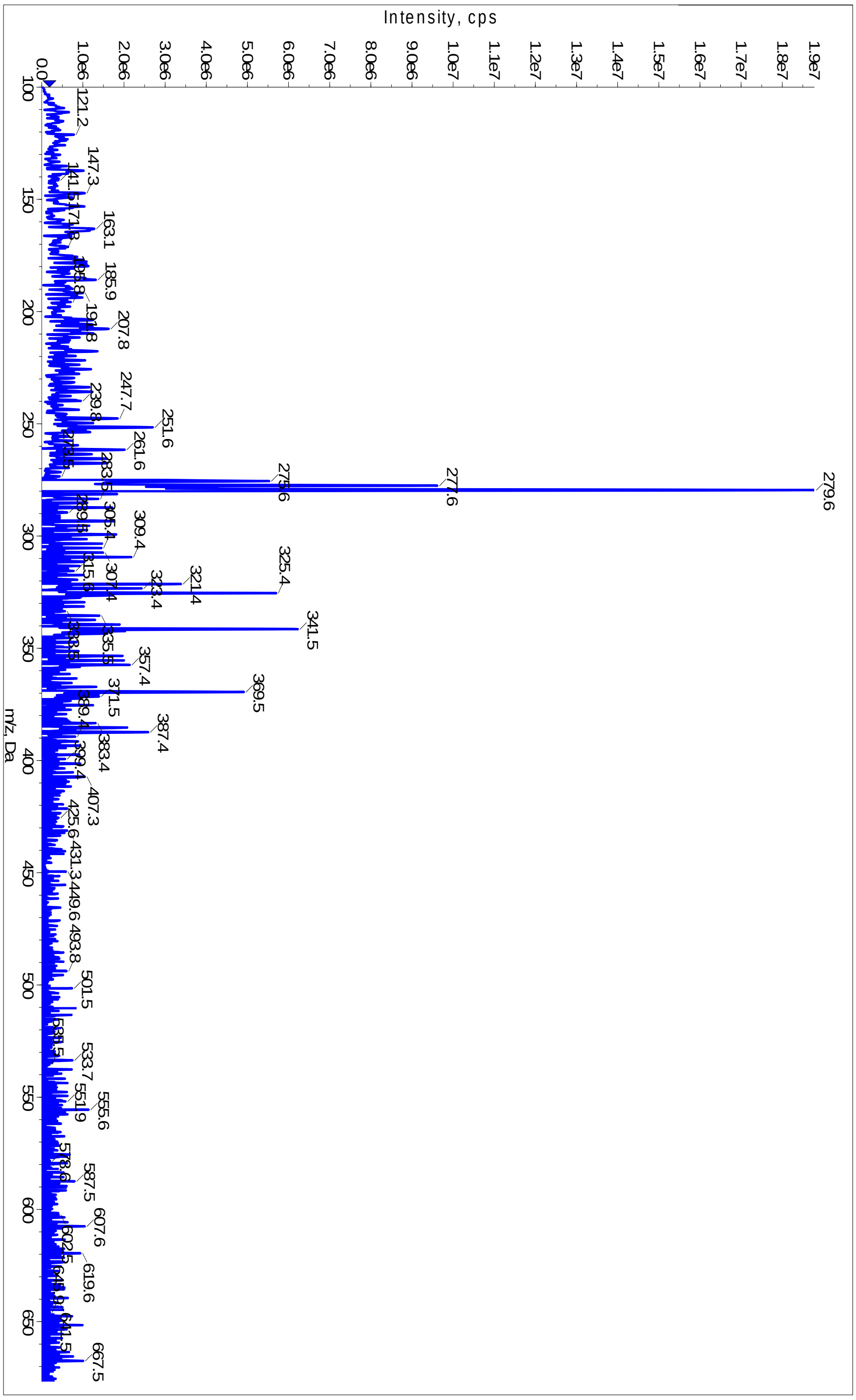

Figure S12. Mass spectrum of 4-epixanthanol (**4**).

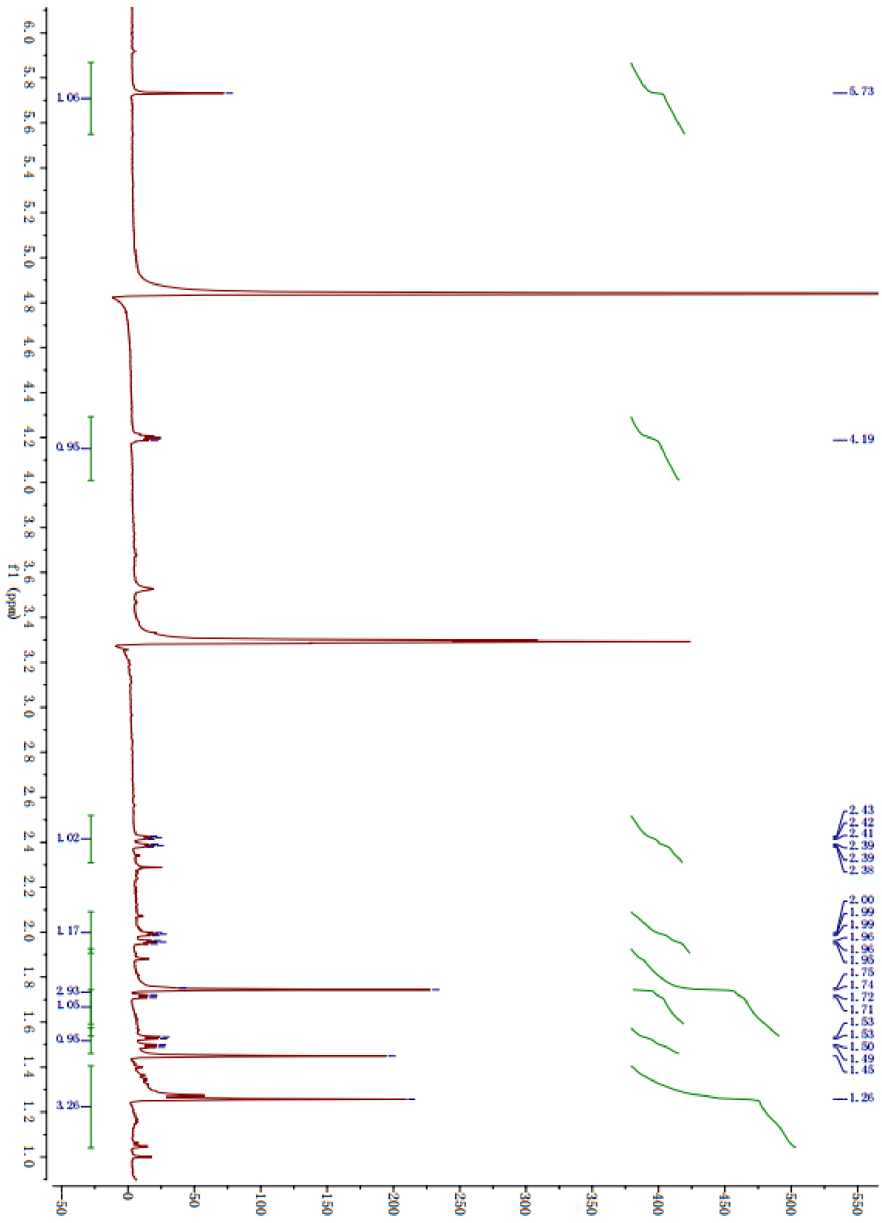

Figure S13. ^{1}H NMR spectrum of loliolide (**5**) (CD_3OD, 400 MHz).

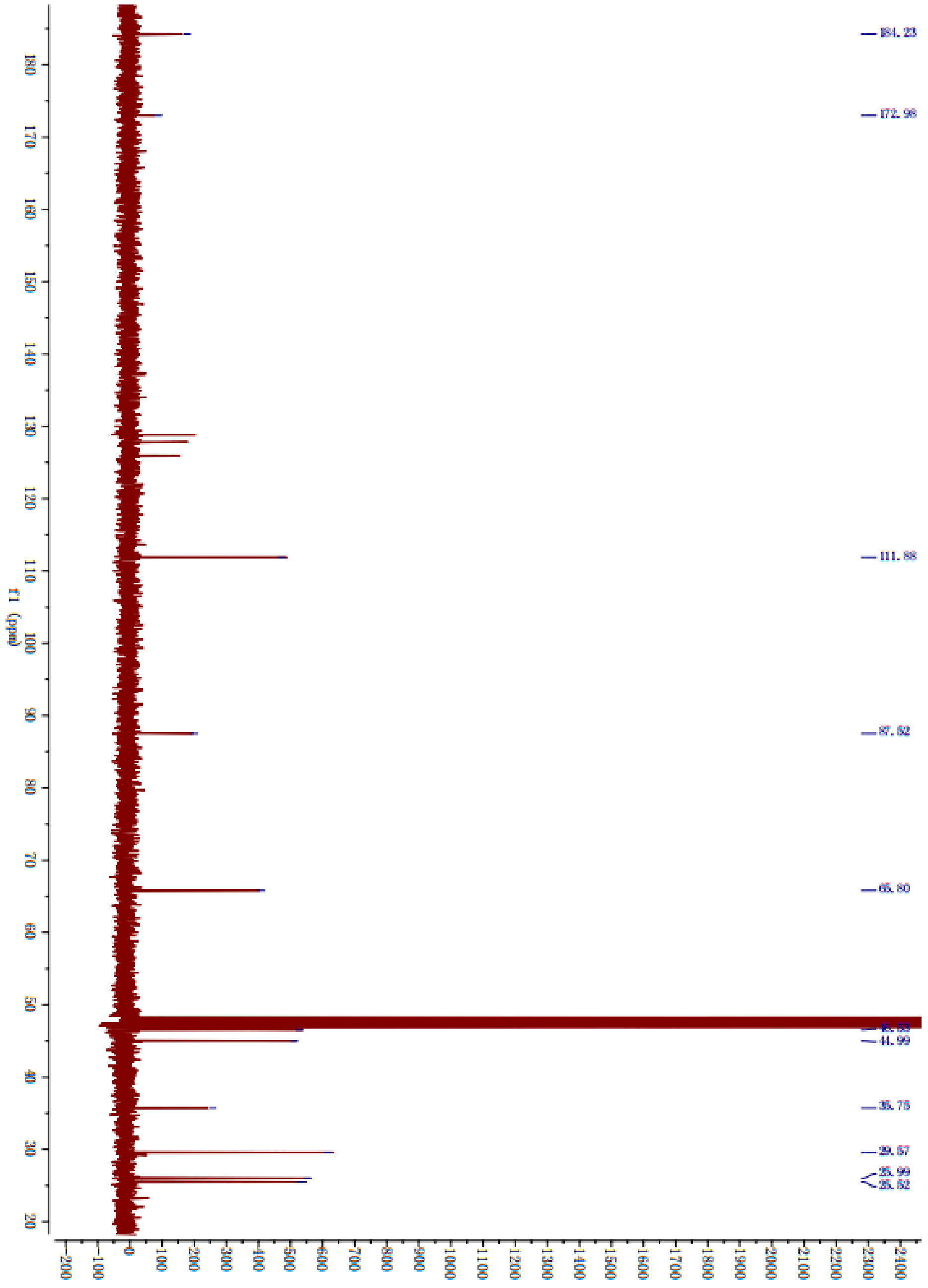

Figure S14. ^{13}C NMR spectrum of loliolide (**5**) (CD_3OD, 100 MHz).

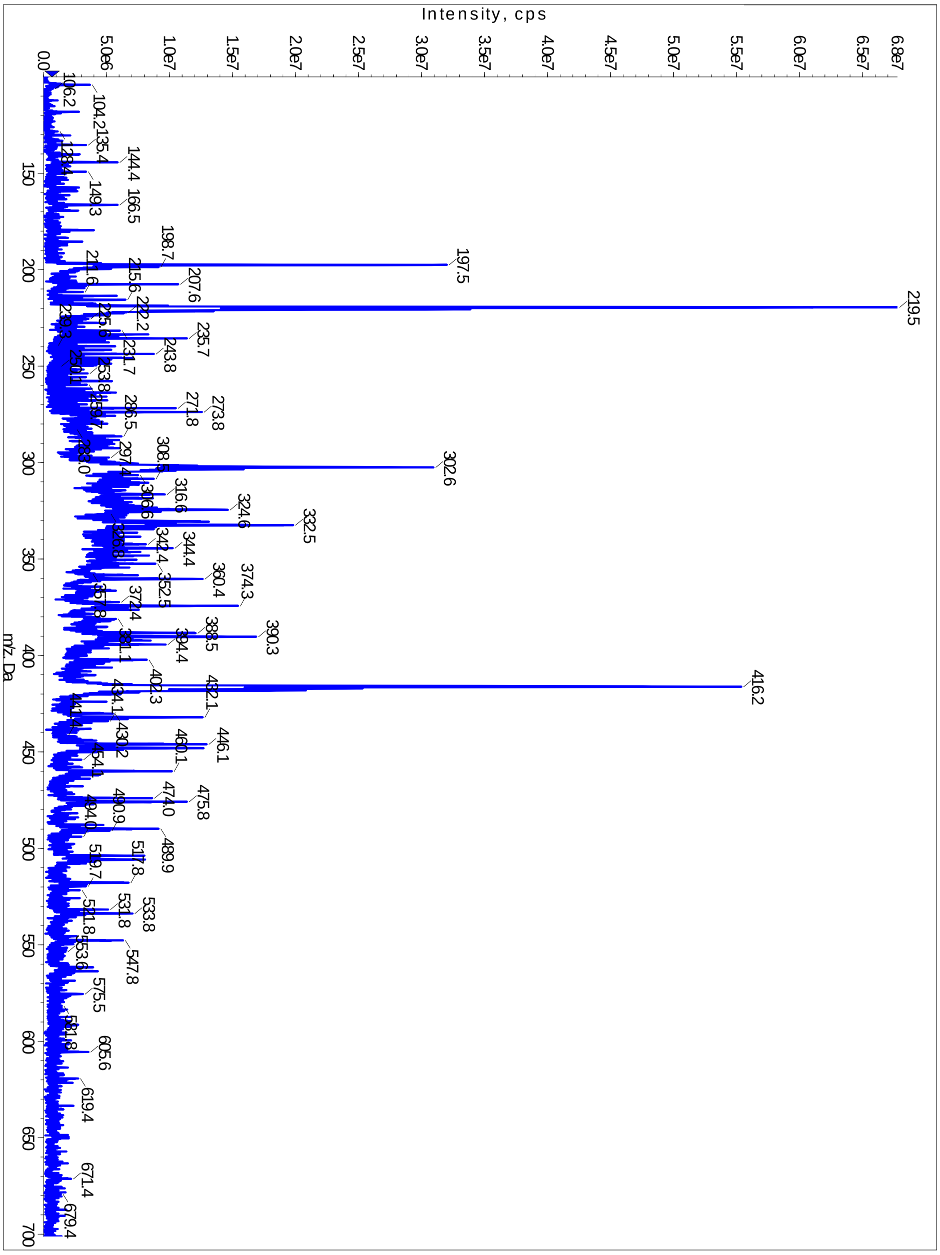

Figure S15. Mass spectrum of loliolide (**5**).

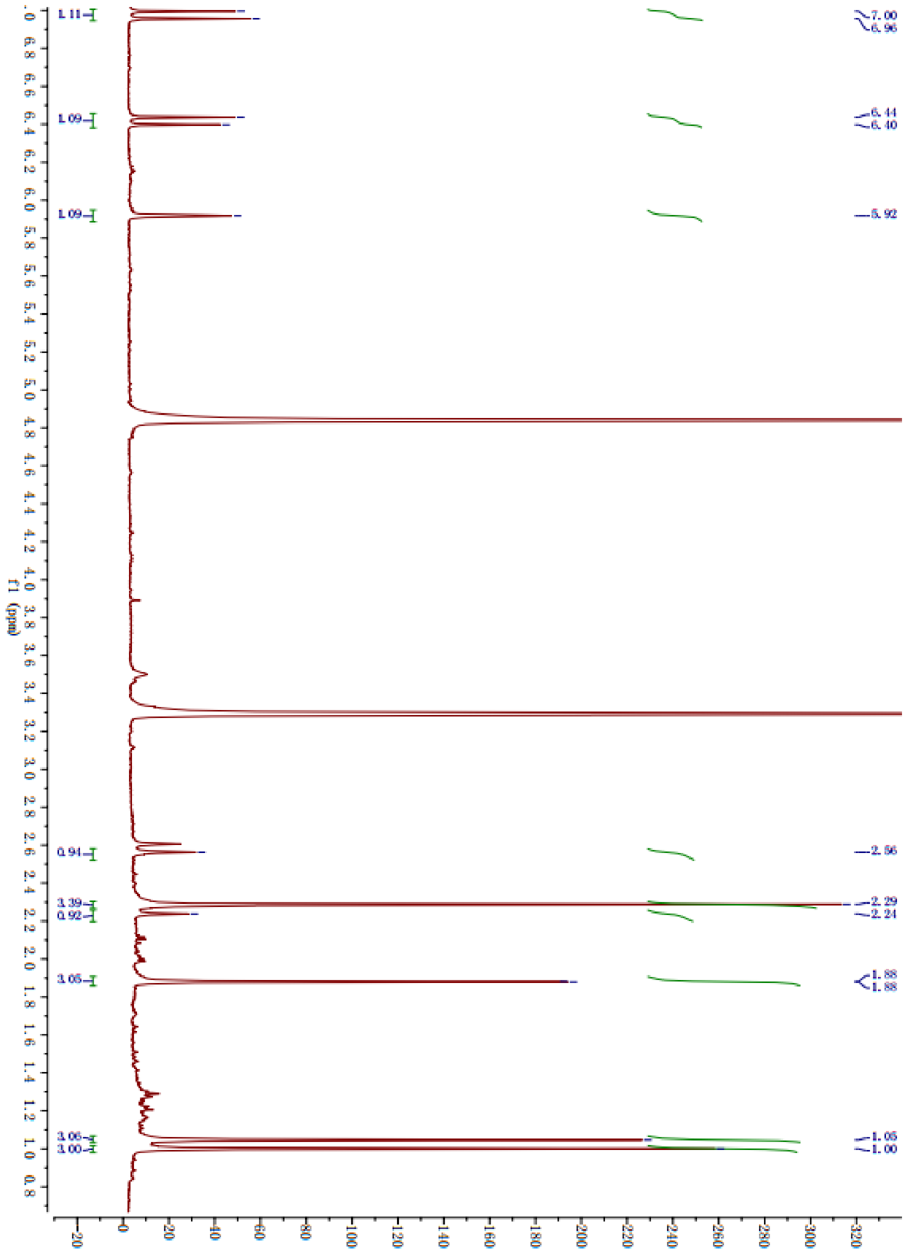

Figure S16. ^{1}H NMR spectrum of dehydrovomifoliol (**6**) (CD_3OD, 400 MHz).

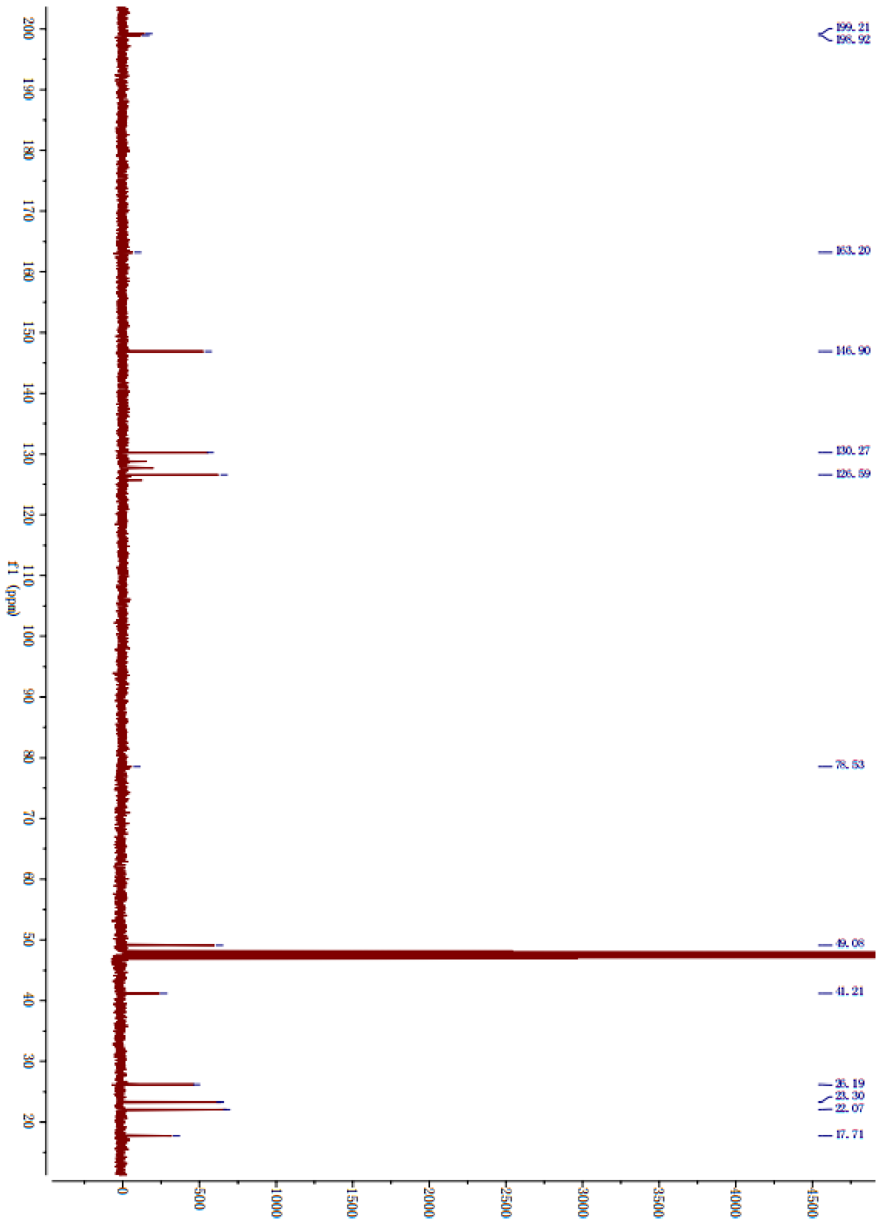

Figure S17. ^{13}C NMR spectrum of dehydrovomifoliol (**6**) (CD_3OD, 100 MHz).

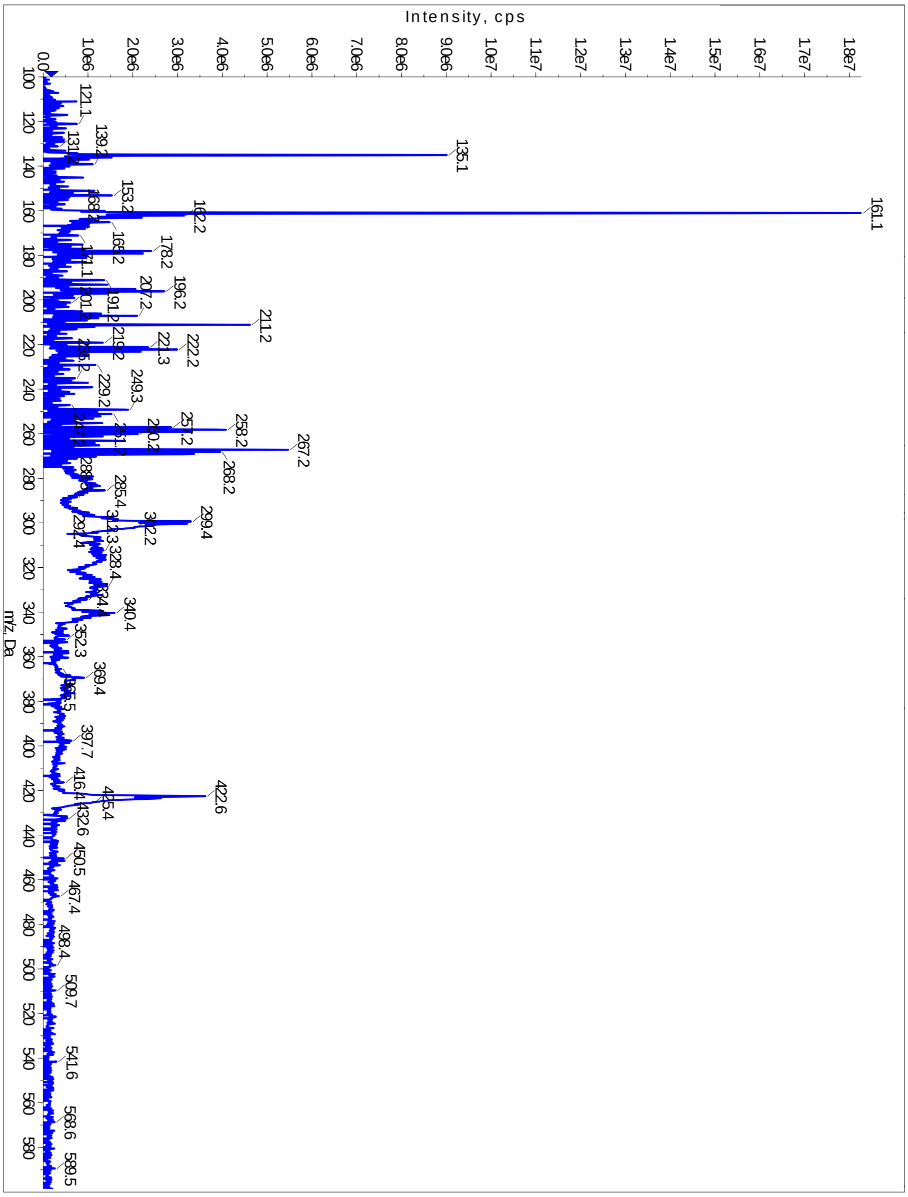

Figure S18. Mass spectrum of dehydrovomifoliol (**6**) .
